# Restriction enzyme digestion of host DNA enhances universal detection of parasitic pathogens in blood via targeted amplicon deep sequencing

**DOI:** 10.1186/s40168-018-0540-2

**Published:** 2018-09-17

**Authors:** Briana R. Flaherty, Eldin Talundzic, Joel Barratt, Kristine J. Kines, Christian Olsen, Meredith Lane, Mili Sheth, Richard S. Bradbury

**Affiliations:** 1 Parasitic Diseases Branch, Division of Parasitic Diseases and Malaria, Centers for Disease Control and Prevention, 1600 Clifton Road, Atlanta, GA 30329 USA; 2 Oak Ridge Institute for Science and Education, 100 ORAU Way, Oak Ridge, TN 37830 USA; 3 Malaria Branch, Division of Parasitic Diseases and Malaria, Centers for Disease Control and Prevention, 1600 Clifton Road, Atlanta, GA 30329 USA; 4 Pacific Biosciences, 1380 Willow Road, Menlo Park, CA 94025 USA; 5 IHRC, Inc., 2 Ravinia Drive, Atlanta, GA 30346 USA; 6 Biotechnology Core Facility, Centers for Disease Control and Prevention, 1600 Clifton Road, Atlanta, GA 30329 USA

**Keywords:** Molecular parasitology, Amplicon sequencing, Blood microbiota, Parasite biodiversity

## Abstract

**Background:**

Targeted amplicon deep sequencing (TADS) of the 16S rRNA gene is commonly used to explore and characterize bacterial microbiomes. Meanwhile, attempts to apply TADS to the detection and characterization of entire parasitic communities have been hampered since conserved regions of many conserved parasite genes, such as the 18S rRNA gene, are also conserved in their eukaryotic hosts. As a result, targeted amplification of *18S rRNA* from clinical samples using universal primers frequently results in competitive priming and preferential amplification of host DNA. Here, we describe a novel method that employs a single pair of universal primers to capture all blood-borne parasites while reducing host *18S rRNA* template and enhancing the amplification of parasite *18S rRNA* for TADS. This was achieved using restriction enzymes to digest the 18S rRNA gene at cut sites present only in the host sequence prior to PCR amplification.

**Results:**

This method was validated against 16 species of blood-borne helminths and protozoa. Enzyme digestion prior to PCR enrichment and Illumina amplicon deep sequencing led to a substantial reduction in human reads and a corresponding 5- to 10-fold increase in parasite reads relative to undigested samples. This method allowed for discrimination of all common parasitic agents found in human blood, even in cases of multi-parasite infection, and markedly reduced the limit of detection in digested versus undigested samples.

**Conclusions:**

The results herein provide a novel methodology for the reduction of host DNA prior to TADS and establish the validity of a next-generation sequencing-based platform for universal parasite detection.

**Electronic supplementary material:**

The online version of this article (10.1186/s40168-018-0540-2) contains supplementary material, which is available to authorized users.

## Background

Several studies have applied next-generation sequencing (NGS) technologies to the investigation of parasite diversity and ecology using various methods to identify all parasites present in a given host [1–5]. Much of this work has depended on metagenomic and metatranscriptomic approaches, including whole genome shotgun sequencing of entire microbial communities [1–3]. Although such approaches are frequently applied to viral and bacterial communities [6–9], direct sequencing of parasite DNA from clinical samples poses challenges with regard to sensitivity and specificity since the concentration of parasite DNA present is often markedly lower in proportion to host DNA. Removal of host DNA via preferential cutting of methylated host sequences using modification-dependent restriction endonucleases has previously been used to increase the recovery of *Plasmodium falciparum* DNA during whole genome sequencing of human blood samples [10]. Unfortunately, this method is only applicable in organisms that do not undergo DNA cytosine methylation, such as apicomplexan parasites [11, 12], and would be ineffective for detecting C5-methylating eukaryotic pathogens [13–15]. Another common approach to increase the capture of parasite DNA relies on using a set of pathogen-specific primers in conjunction with a strand-displacing DNA polymerase to achieve selective whole genome amplification [16, 17]. However, species-specific methods such as this are difficult to adapt to broader analyses of whole parasite communities.

Over the past decade, targeted amplicon deep sequencing (TADS) of the 16S rRNA gene has frequently been used to study and characterize bacterial microbiomes [18–22]. A similar approach, using universal PCR primers to target a conserved parasite gene for TADS, would be amenable to studies of parasite communities. Unfortunately, such an approach is usually compromised by the overabundance of host DNA, as common primer targets are conserved across higher order eukaryotic species, including metazoan parasites. A recent study sought to overcome this challenge by utilizing host DNA blocking primers in the assessment of parasite biodiversity in the feces of wild rats [5]. However, this method was rarely able to achieve species-level identification, and application of the method to assess helminth biodiversity ultimately required worm isolation from fecal samples and amplification with class-specific primers [4].

To overcome the challenge of host DNA interference, we designed a TADS method that utilizes restriction enzymes to reduce amplification of host DNA template prior to PCR and NGS. Using universal primers to target the 18S rRNA gene in a region containing restriction enzyme cut sites only present in the host sequence, host *18S rRNA* template was digested, and PCR enrichment of the host sequence was reduced to allow enhanced detection of parasite *18S rRNA*. This method achieved a substantial reduction in reads belonging to the host and a 5- to 10-fold increase in parasite reads following NGS. The method was validated using 16 species of human blood-borne parasites and was effective in detecting both single and mixed parasite infections. Limit of detection analyses showed consistent reduction in LOD for digested versus undigested samples, where positive results were achieved for specimens with parasitemias as low as ~ 7 parasites per microliter for digested samples versus a low of ~ 40 parasites per microliter for undigested samples. This method provides a single assay for detection of all major blood-borne parasites found in humans and represents a promising new tool for the study of parasite communities.

## Results

### Assay design

**Fig. 1:**
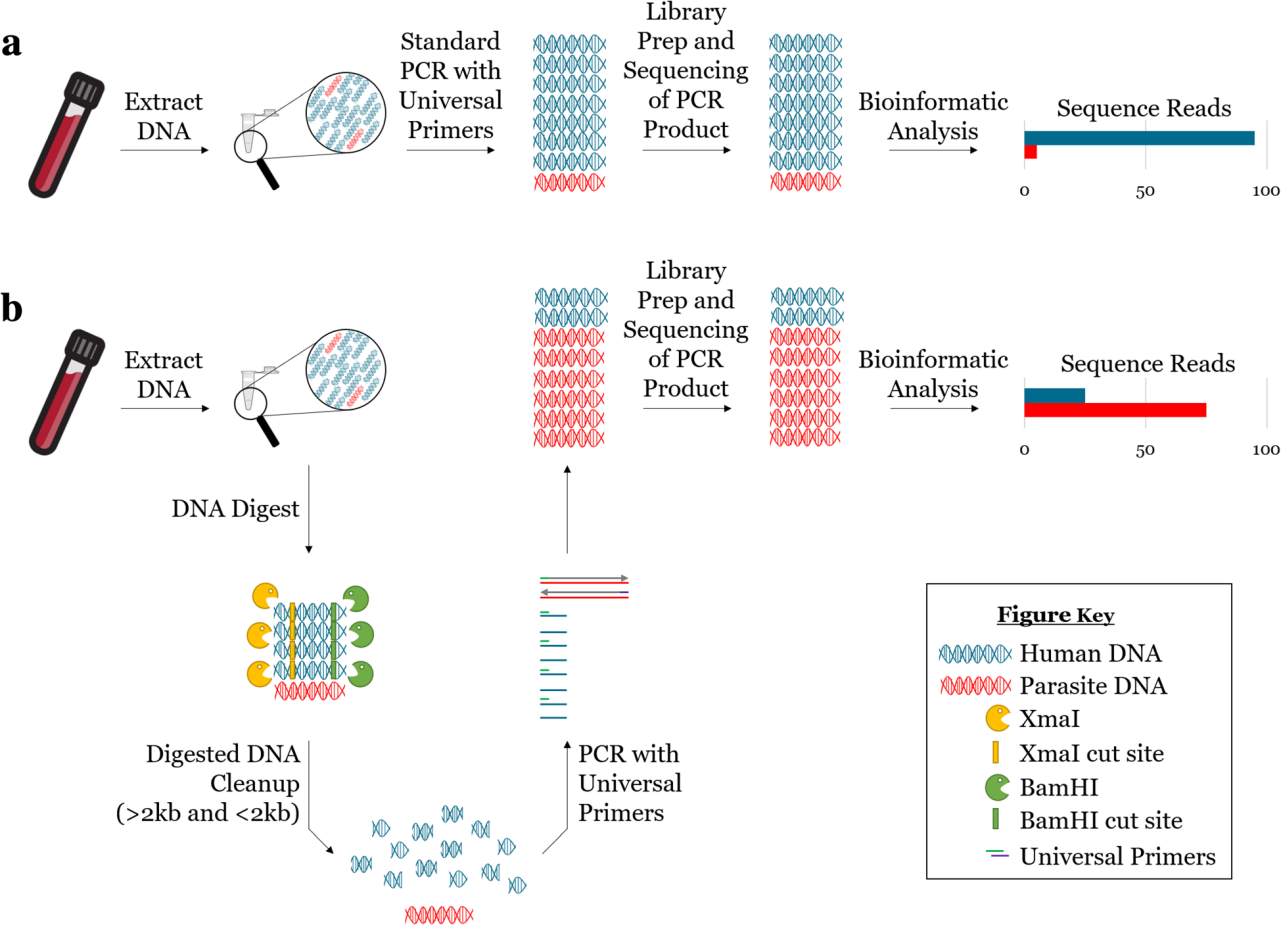
Reduction of host DNA by restriction enzyme digestion enhances PCR amplification of parasite DNA. DNA extraction from parasite-infected whole blood yields a DNA sample containing high amounts of host DNA (blue) and low amounts of parasite DNA (bright red). **a** Performing conventional PCR on this sample, using universal primers, amplifies primarily host DNA (blue), and yields sequencing reads almost entirely belonging to the host. **b** In contrast, restriction enzyme digestion of host DNA prior to PCR alters the ratio of host to parasite DNA in the initial sample, allowing for selective amplification of parasite DNA (bright red) and resulting in an increase in the relative number of parasite amplicons post-PCR and an increase in the sensitivity of parasite detection via NGS

Primers were designed to amplify a region of the 18S rRNA gene approximately 200 base pairs in length that is highly conserved across eukaryotic organisms yet contains sufficient diversity at the nucleotide level to allow accurate species identification and differentiation. The selected amplification region possesses BamHI and XmaI restriction enzyme cut sites only in the human host sequence (Additional file 1: Figure S1), which allows cleavage of host template and reduced amplification of host DNA during PCR amplicon enrichment prior to Illumina amplicon deep sequencing (Fig. 1). Following sequencing, paired and trimmed reads were mapped to a database of human and parasite *18S rRNA* sequences, and the number of mapped reads per parasite species was counted. Given the sensitivity of Illumina sequencing, the occurrence of Illumina index cross-talk, and because some DNA cross-contamination can be expected in samples that are extracted and processed together [23–25], we established a conservative, dual-criterion system to differentiate “noise” from a true positive sequencing result. This system utilizes a minimum cutoff for positivity based on the average proportion of contaminating reads obtained per negative control specimen over multiple replicate analyses (assuming 60–80 samples are multiplexed in a single library, see the “Methods” section for further details). In addition, a species-specific shifting maximum cutoff value was established to be applied on a per specimen basis and to account for minor changes in the degree of index cross-talk and variations in the number of reads generated per specimen/experiment. Using this dual-criterion system, specimens were considered positive only if more than 20 reads mapped to the respective parasite reference sequence and if the number of parasite derived reads mapped to that reference sequence also exceeded the shifting maximum cutoff value.

Since total sequencing reads can vary from run to run, results were normalized to allow comparisons between experiments. Reads were normalized according to the total number of paired reads per sample (after trimming) and reported as reads per thousand. To assess the impact of restriction enzyme reduction of competitive host template DNA and the capacity to detect a variety of blood parasites, this technique was applied to clinical blood specimens with and without prior restriction endonuclease treatment. For these experiments, paired DNA specimens (i.e., restriction digested specimens and their respective undigested partner) were sequenced in the same Illumina library so that direct comparisons could be made between the two conditions.

### Assay validation

Human or (surrogate) animal blood samples containing previously diagnosed parasitic infections (Table 1) were processed in triplicate, as shown in Fig. 1b. Bioinformatic analysis indicated that the primer binding sites, the region of amplification, and the restriction enzyme cut sites of all relevant animals share 100% identity with the human target sequence. Consequently, samples containing animal blood were analyzed in an identical fashion to human clinical samples. Most blood-borne parasites known to infect humans were included in our analysis; however, we were unable to obtain clinical samples/isolates for *Trypanosoma brucei* subsp. *gambiense* and several rare human filarial blood parasites*.* Bioinformatic analysis of the complete *18S rRNA* sequence from several blood parasites identified BamHI and/or XmaI cut sites outside the region of amplification, varying slightly in their location and frequency between parasite taxa. As such, the resulting restriction fragments would vary in size for different blood parasites. Consequently, post-digestion DNA for all validations was divided into two equal parts and cleaned using a Monarch PCR & DNA Cleanup Kit selecting for both > 2 kb and < 2 kb DNA products, respectively. Prior to DNA extraction and restriction digestion, all samples were spiked with cat blood containing 3.4 × 10^6^ *Cytauxzoon felis* parasites as an extraction, amplification, and sequencing internal control.

**Table 1:** Host, source, original identification method and GenBank accession number of samples used in this study

Parasite	Sample type	Host	Species identification diagnostic method/s	DNA extraction method	Source	GenBank accession no.
*Babesia divergens*	EDTA blood	*Meriones unguiculatus*	Microscopy and PCR [35] w/s	Qiagen Blood Mini Kit	Henry Bishop, CDC PDB	AJ439713
*Babesia duncani*	EDTA blood	*Meriones unguiculatus*	Microscopy and PCR [35] w/s	Qiagen Blood Mini Kit	Henry Bishop, CDC PDB	HQ289870
*Babesia microti*	EDTA blood	*Homo sapiens*	Microscopy and PCR [35] w/s	Qiagen Blood Mini Kit	Henry Bishop, CDC PDB	AB243680
*Brugia malayi*	EDTA blood	*Felis catus*	Microscopy	Qiagen Blood Mini Kit	Andrew Moorhead, FR3	AF036588
*Cytauxzoon felis*	EDTA blood	*Felis catus*	Microscopy	Qiagen Blood Mini Kit	David Peterson, UGA	CXZRR18S
*Leishmania donovani* subsp. *donovani*	RPMI culture in EDTA blood	*Homo sapiens*	Microscopy and PCR [36] w/s	Qiagen Blood Mini Kit	Marcos de Almeida, CDC PDB	X07773
*Leishmania donovani* subsp. *infantum*	RPMI culture in EDTA blood	*Homo sapiens*	Microscopy and PCR [36] w/s	Qiagen Blood Mini Kit	Marcos de Almeida, CDC PDB	GQ332359
*Loa loa*	EDTA blood	*Homo sapiens*	Real time PCR [37]	Boiled lysate	Thomas Nutman, NIH	DQ094173
*Mansonella perstans*	EDTA blood	*Homo sapiens*	Microscopy	Boiled lysate	Thomas Nutman, NIH	–
*Plasmodium falciparum*	EDTA blood	*Homo sapiens*	Microscopy and PCR [38] w/s	Qiagen Blood Mini Kit	Henry Bishop, CDC PDB	PFARGEA
*Plasmodium knowlesi*	EDTA blood	*Macaca mulatta*	Microscopy	Qiagen Blood Mini Kit	Amy Kong, CDC Malaria Branch	PFARRSSU
*Plasmodium malariae*	EDTA blood	*Homo sapiens*	Microscopy and PCR [38] w/s	Qiagen Blood Mini Kit	Henry Bishop, CDC PDB	PFARGBAB
*Plasmodium ovale*	EDTA blood	*Homo sapiens*	Microscopy and PCR [38] w/s	Qiagen Blood Mini Kit	Henry Bishop, CDC PDB	KF696369
*Plasmodium vivax*	EDTA blood	*Homo sapiens*	Microscopy and PCR [38] w/s	Qiagen Blood Mini Kit	Henry Bishop, CDC PDB	X13926
*Tryapnosoma cruzi*	RPMI culture in EDTA blood	*Homo sapiens*	Microscopy	Qiagen Blood Mini Kit	Marcos de Almeida, CDC PDB	AF239980
*Trypanosoma brucei* subsp. *gambiense*	–	–	–	–	–	–
*Trypanosoma brucei* subsp. *rhodesiense*	HMI-9 culture in EDTA blood	*Homo sapiens*	Microscopy	Qiagen Blood Mini Kit	Stephen Hajduk, UGA	AJ009142
*Wuchereria bancrofti*	Clotted blood	*Homo sapiens*	Microscopy	Qiagen Blood Mini Kit	Patrick Lammie, CDC PDB	AF227234

*w/s* With Sanger sequencing of PCR product; *CDC* Centers for Disease Control and Prevention; *PDB* Parasitic Diseases Branch; *UGA* University of Georgia; *NIH* National Institutes of Health

**Fig. 2:**
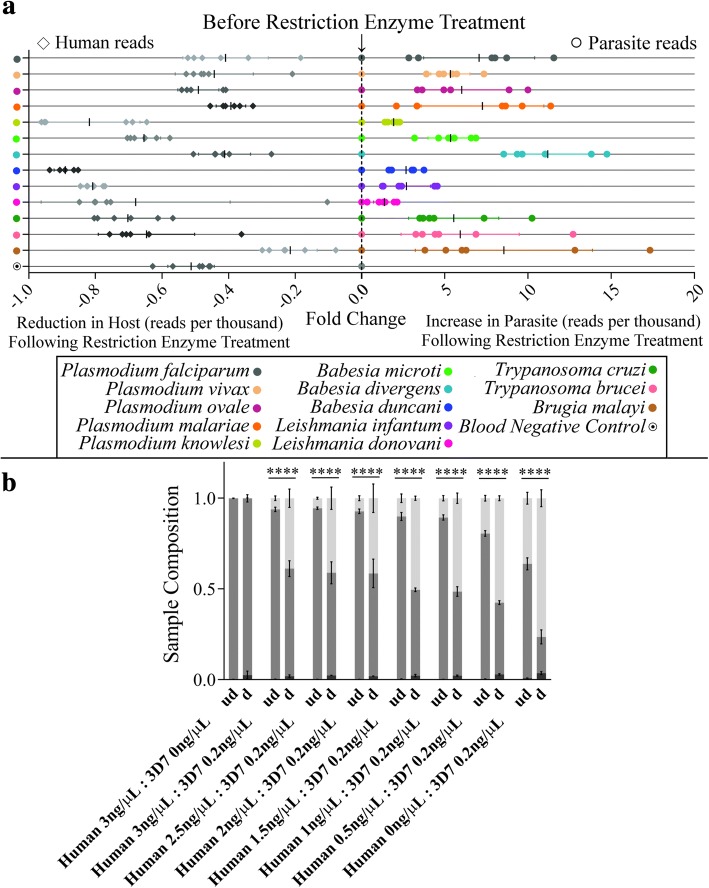
Digestion of host DNA increases the sensitivity of parasite detection in parasite-positive human blood samples. (**a**) Restriction enzyme digestion yields a marked reduction in human *18S rRNA* reads per thousand (left panel, greyscale diamonds) and a 5- to 10-fold increase in parasite reads per thousand (right panel, colored circles) in digested relative to undigested samples (*n* = 3 biological replicates, mean ± SD, samples were normalized according to the reads per thousand for reads derived from human host and parasite separately, with the central dotted line reflective of a zero fold change, which marks the undigested samples before treatment with restriction enzymes). No statistical difference was found for size selection (i.e., > 2 kb vs. < 2 kb) (two-way ANOVA, *p* = 0.0631). (**b**) Proportional composition of human DNA dilutions in undigested (ud) and digested (d) samples demonstrates an average 2-fold reduction in human DNA and a 5-fold increase in parasite reads post-digestion (black bars = *C. felis*, dark grey bars = H. sapiens, light grey bars = *P. falciparum*, concentration of 3D7 DNA includes *P. falciparum* and *H. sapiens* DNA from 3D7 cultures which contain human blood products, two-way ANOVA with Sidak’s multiple comparisons posttest, *p* < 0.0001, *n* = 3, mean ± SD)

Following TADS, a substantial reduction in reads mapping to the human host reference was observed in the digested samples compared to undigested samples (Fig. 2a). Furthermore, the digested samples showed a 5- to 10- fold increase in the number of parasite-specific reads (Fig. 2a and Additional file 2: Figure S2). All samples assayed passed our set criteria for positivity except for two *Wuchereria bancrofti* blood samples. For these samples, the failure to detect *W. bancrofti* was attributed to the formation of a large, non-uniform clot that made DNA extraction problematic (Additional file 3: Figure S3a-c). Nevertheless, these data suggest that reduction of host DNA background via restriction enzyme digestion improves detectability of parasite DNA for the universal detection of parasites in blood. Analysis of post-digestion size selection found no statistical difference between > 2 kb and < 2 kb cleanup conditions (*p* = 0.0631).

### Digestion reduces host reads by 50% or more

To determine the extent to which enzyme digestion reduces human host background, a series of dilutions was prepared using human DNA donated by healthy volunteers and parasite DNA obtained from a 3D7 *P. falciparum* culture. Samples contained 0.2 ng/μL *P. falciparum* DNA, or approximately 8600 parasites per microliter, as well as human DNA diluted to a concentration of 3, 2.5, 2, 1.5, 1, 0.5, or 0 ng/μL. Bioinformatic analysis indicated no BamHI/XmaI cut sites within 2 kbp of the PCR amplification region for *P. falciparum* 3D7, so all post-digestion samples were cleaned according to the > 2 kb size selection protocol. In undigested samples, 80–100% of sequencing reads mapped to the human host reference sequence with only 0–20% of reads mapping to *P. falciparum* (Fig. 2b). Meanwhile, the composition of digested sample reads was only 40–50% human and 50–60% *P. falciparum*, reflecting a greater than or equal to 2-fold decrease in human reads and an average 5-fold increase in parasite reads post-digestion (Fig. 2b).

As an additional control, a mock restriction digestion was performed in triplicate wherein DNA extracted from human blood spiked with cultured 3D7 parasites was incubated for the same period and at the same temperature as identical specimens subjected to a true restriction enzyme digestion. These samples were subsequently purified using a Monarch Cleanup Kit (> 2 kb) and PCR amplified according to the protocol described. Post-sequencing analysis found no statistical difference in the number of 3D7-derived sequencing reads obtained between mock digested DNA specimens and their paired undigested DNA samples, confirming that the increase in parasite reads in the restriction-digested samples is directly related to the action of the restriction enzymes on host DNA (Additional file 4: Figure S4).

### Detection of mixed parasite infections

**Fig. 3:**
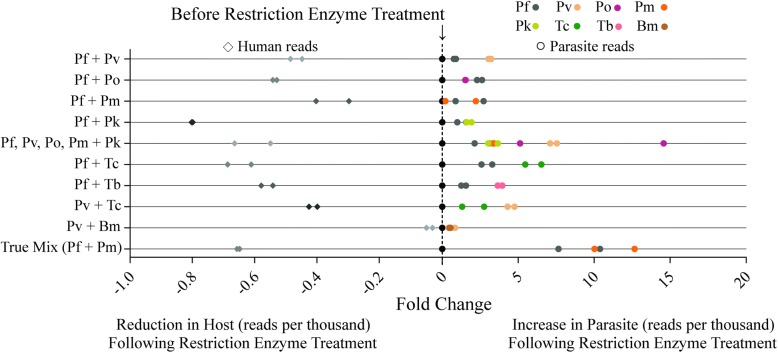
Enzyme digestion enhances sensitivity of detection for mixed parasite infections. Restriction enzyme digestion of mixed parasite infections in human blood yields a clear reduction in human *18S rRNA* reads per thousand (left panel, greyscale diamonds) and a 2- to 15-fold increase in parasite reads per thousand (right panel, colored circles). Samples were normalized according to the reads per thousand for reads derived from human host and parasite separately, with the central dotted line reflective of a zero fold change, which marks the undigested samples before treatment with restriction

To explore the effectiveness of this method for detecting mixed infections, a variety of mixed parasite blood samples were artificially produced by combining previously diagnosed parasite-infected blood samples and subjecting them to analysis via the described method. The samples simulated all varieties of mixed malaria infections, including all the major *Plasmodium* species that infect humans, together and in pairs, as well as other geographically possible mixed infections. As before, we saw a dramatic decrease in human reads in digested relative to undigested samples and, in this case, a 2- to 15-fold increase in parasite reads post-digestion (Fig. 3, left panel). These data establish that this method can reliably detect parasites in mixed infections but suggest that competitive amplification occurs between the different *18S rRNA* types, which may affect the sensitivity of detection for parasite species occurring at relatively low numbers in mixed parasite communities.

To further assess assay effectiveness in detecting mixed infections, a non-artificial (natural) mixed malaria infection that had been previously diagnosed by the CDC Parasite Reference Diagnostic Laboratory was tested. Interestingly, in this case, enzyme digestion proved to be the deciding factor between an accurate and inaccurate assessment of the sample by NGS. While universal PCR and NGS of the undigested sample showed positive results for only *P. falciparum*, sample digestion led to a greater than 10-fold increase in aligned reads for *Plasmodium malariae*, a more accurate evaluation of this mixed parasite community (Fig. 3, right panel).

### Digestion improves the limit of detection of parasites in blood

**Fig. 4:**
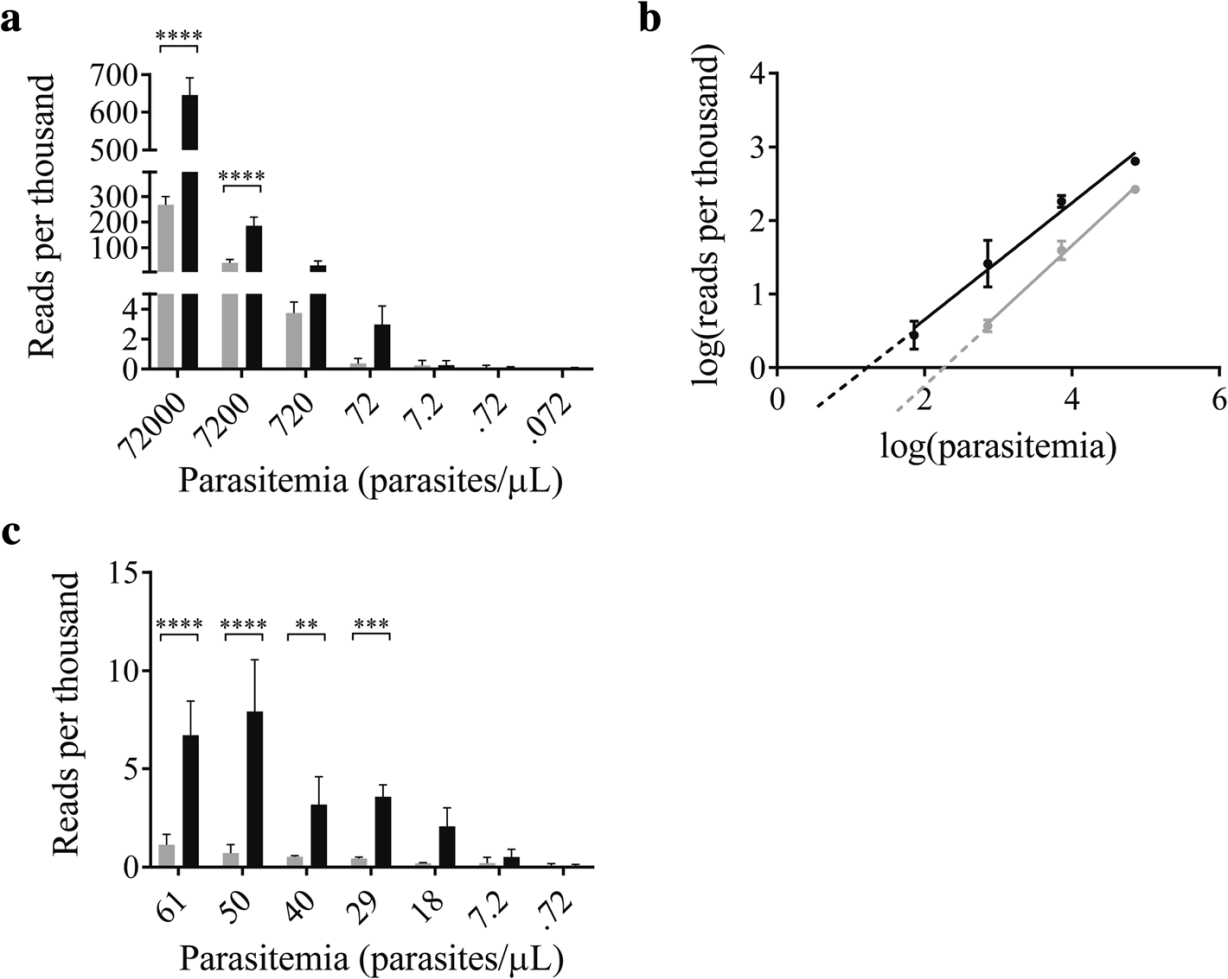
Enzyme digestion markedly lowers assay limit of detection. (**a**) Reads per thousand for undigested (gray) and digested (black) 10-fold serial dilutions of *P. knowlesi* in whole human blood (*n* = 4, mean ± SD). (**b**) Log-transformation of reads per thousand from serially diluted samples suggests a limit of detection of 163 parasites per microliter for undigested samples (gray, r^2^ = 0.9852) and 15 parasites per microliter for digested samples (black, *r*^2^ = 0.9533) (*n* = 4 biological replicates, mean ± SD). (**c**) After deeper analysis, reads per thousand for undigested (gray) and digested (black) serial dilutions between 61 parasites per microliter and 0.72 parasites per microliter demonstrate a limit of detection of 40 to 60 parasites per microliter for undigested samples and 7 to 29 parasites per microliter for digested samples (two-way ANOVA with Sidak’s multiple comparisons posttest, **** *p* < 0.0001, *** *p* < 0.001, ** *p* < 0.005 *n* = 3, mean ± SD)

To quantify the extent to which enzyme digestion improves the limit of detection (LOD) for this method, *Plasmodium knowlesi*-infected rhesus macaque blood with 3.3% parasitemia (approximately 144,000 parasites per microliter) was utilized. Three aliquots of the sample were serially diluted in parasite-negative whole human blood to a parasitemia of 0.072 parasites per microliter and analyzed in biological triplicate. For the region amplified, the rhesus macaque and human *18S rRNA* target sequences are identical, and thus, sample digestion will be equivalent despite the host species being different. Post-digestion samples were, again, cleaned only according to the > 2 kb size selection protocol. As expected, the LOD for samples that had not undergone enzyme digestion before PCR and sequencing was high, such that reads began to fall below baseline at parasitemias between 720 and 72 parasites per microliter; meanwhile, prior enzyme digestion caused samples to fall below baseline between 72 and 7.2 parasites per microliter (Fig. 4a). A trend line was fitted to the log-transformed data to determine a more precise LOD before and after enzyme digestion (Fig. 4b). Using this trend line, the LOD for undigested samples was extrapolated to 163 parasites per microliter while that of the digested samples was estimated at 15 parasites per microliter. As this estimate was extrapolated from a trend line rather than empirical data, a series of finer dilutions (between 61.2 and 0.72 parasites per microliter) was performed to establish a more precise LOD (Fig. 4c). With restriction enzyme digestion, it was confirmed that the assay can detect as few as 7.2 *P. knowlesi* parasites per microliter of blood, albeit inconsistently, as this LOD was achieved for only two out of three triplicate samples with the third replicate detecting only 28.8 parasites per microliter (Table 2). For the identical set of undigested specimens, a consistent positive result was obtained only at the highest parasite concentration of 61.2 parasites per microliter, with one replicate detecting down to 39.6 parasites per microliter and a second detecting only 50.4 parasites per microliter. Based on this data, there is a 1.4- to 8.5-fold improvement in LOD following restriction enzyme digestion.

**Table 2:** Detection of *P. knowlesi* in blood at different concentrations following restriction enzyme treatment of DNA extracts

	*Pk* reads/total number of reads			Result		
*Pk* parasites/μL	R1	R2	R3	R1	R2	R3
61.2	$\frac{54}{10994}$	$\frac{383}{45566}$	$\frac{208}{30568}$	+	+	+
50.4	$\frac{44}{6160}$	$\frac{180}{31360}$	$\frac{305}{28068}$	+	+	+
39.6	$\frac{31}{7562}$	$\frac{231}{47096}$	$\frac{44}{28514}$	+	+	+
28.8	$\frac{26}{9030}$	$\frac{84}{21680}$	$\frac{95}{23942}$	+	+	+
18	$\frac{18}{12074}$	$\frac{24}{15722}$	$\frac{62}{19654}$	–	+	+
7.2	$\frac{12}{20290}$	$\frac{22}{19068}$	$\frac{43}{63164}$	–	+	+
0.72	$\frac{2}{36190}$	$\frac{2}{17220}$	$\frac{6}{57076}$	–	–	–

*Pk Plasmodium knowlesi*, *R* replicate, *+* positive, *−* negative

Note: The read counts listed here represent the values obtained after trimming and filtering

## Discussion

Potential applications of this method extend beyond detection of parasitic pathogens in human blood. This method may allow for exploration of mammalian blood parasites for ecological, wildlife disease, zoonotic disease, and pathogen discovery studies. To explore these potential applications, the 18 s rRNA sequences of various classes of vertebrates relative to the human target DNA sequence were analyzed in silico. Both the BamHI and XmaI restriction enzyme cut sites, the PCR primer binding sites, and in some cases, the entire amplification region were conserved in mammals and birds (Additional file 5: Figure S5 and Additional file 6: Table S1). Among the vertebrates analyzed were many common livestock and companion animals. Within the region of amplification, these animals shared greater than 98% identity with the human sequence, suggesting this methodology may be applied in animal hosts for agricultural and veterinary purposes.

It is also pertinent to consider the effectiveness of this method in exploring the mycobiome. A preliminary bioinformatic analysis of DNA sequences from several common fungal organisms was conducted and found that neither the BamHI nor the XmaI cut sites were present in any fungi tested while primer binding sites were universally conserved (Additional file 7: Figure S6 and Additional file 8: Table S2). It may be inferred from these data that this platform will provide a tool for the detection and identification of fungal infections in eukaryotic hosts, as well. Further investigation and validation of these additional applications is underway.

A recent study evaluating the LOD for several published *Plasmodium* species’ real time PCR (qPCR) assays reported LODs in the range of 0.3 to 2.5 parasites per microliter [26]. Conventional PCR and loop-mediated isothermal amplification (LAMP) assays are generally less sensitive than qPCR, with LODs usually falling between 1 and 20 parasites per microliter of blood [27, 28]. The LOD of the assay described herein falls between 7 and 29 parasites per microliter (Table 2) and is therefore similar to those reported for conventional PCR assays. Rapid diagnostic tests for detection of malaria antigens are typically less sensitive and are reportedly most suitable for detecting parasitemias above 200 parasites per microliter [29]. Meanwhile, for *Plasmodium* species, it is estimated that a highly competent microscopist can detect approximately 50 parasites per microliter of blood [30] while a typical microscopist using the WHO standardized method detects an average of 88 parasite per microliter [31]. Thus, the method described herein possesses an LOD for *Plasmodium* species similar to published conventional PCR assays, but boasts the added advantage of being able to detect and identify all human blood-borne parasitic pathogens in a sample using a single test.

An important consideration regarding the sensitivity of this assay is the high amount of variation in *18S rRNA* copy number between different species of blood parasites. For example, the rDNA copy number of *P. falciparum* ranges from five to eight copies per haploid genome [32]. Other apicomplexan blood parasites possess a similarly low copy number, such as *Plasmodium vivax* which possesses four to eight copies [33] and *Babesia microti* with only two copies of rDNA [34]. It is reasonable to assume that this method will have increased sensitivity for parasite species with higher rDNA copy numbers, such as *T. brucei* (56 copies), *Trypanosoma cruzi* (110 copies) and *Leishmania donovani* (166 copies) [33]. Further investigation will be required to establish limits of detection for other parasite species.

Multiple non-protozoan blood parasites were used to validate this methodology for universal detection of parasites in blood. Several challenges were encountered due to the very limited availability of these parasites; in these cases, the few samples that were available were tested. For example, the results obtained for *W. bancrofti* analysis were skewed as only a clotted blood sample, which is not the recommended matrix for this test, was available. This led to an uneven distribution of parasites and subsequent inconsistencies in parasite DNA concentration between blood samples (Additional file 3: Figure S3a). Nevertheless, results of host DNA digestion in clotted samples remained consistent at 1.5- to 2.5-fold reduction in human reads (Additional file 3: Figure S3b), and results for *W. bancrofti* were usually positive, despite the observed variations (Additional file 3: Figure S3c). The *Loa loa* sample sequenced was DNA extracted from an adult worm, and thus, we were unable to consider reductions in human DNA for that parasite. The resultant sequencing reads from this parasite sample were consistently high (Additional file 3: Figure S3d), and no change was observed between digested and undigested samples due to the lack of human DNA background (Additional file 3: Figure S3e). Although processing of boiled lysates of human blood infected with *Mansonella perstans* yielded an appropriately sized PCR product on agarose gel electrophoresis as well as greater than 50,000 paired reads following NGS, it was not possible to confirm the identity of this species as no reference *M. perstans* *18S rRNA* sequence for the region amplified is currently available in any genome database. However, a large number of unused paired reads (10,000–15,000) from the raw sequences following reference alignment to the human *18S rRNA* reference suggested that a large amount of non-host eukaryotic DNA had been amplified in this sample. Further investigation revealed that 10,298 of these reads aligned with 100% identity to a sequence in GenBank designated as an *18S rRNA* sequence from a *Filarioidea* sp. (accession: KT907503.1). It is, therefore, proposed that these reads are likely derived from *M. perstans* DNA amplified from within that sample.

A limitation of this method is that it does not amplify regions with sufficient sequence variation to differentiate *W. bancrofti* from *L. loa* or to discriminate between some parasite subspecies, such as *T. brucei* subsp. *gambiense* and *T. brucei* subsp. *rhodesiense* or *L. donovani* subsp. *donovani* and *L. donovani* subsp. *infantum*. The demands of identifying a single region flanked by universally conserved primer-binding sites and possessing restriction enzyme recognition sites that cut only the host gene, was unfortunately restricting. However, infections with these agents are rare in most parts of the world and differentiation when such cases are detected may be undertaken by a further, species-specific PCR.

## Conclusions

This universal detection platform offers a versatile and broad-spectrum method for the study of parasitic communities in human hosts. Improved sensitivity was obtained by employing restriction enzymes targeting host-specific cut sites to selectively limit the amplification of unwanted host DNA sequences and reduce competitive PCR amplification of host *18S rRNA*. This method has been validated in biological triplicate for 16 human blood-borne parasites. Future exploration and optimization of this method for the study of parasite diversity and the detection of parasitic disease in other eukaryotic hosts, as well as in other sample matrices such as tissue and feces, is warranted.

## Methods

### Samples

Human clinical blood samples used in this study were originally submitted to the CDC Parasitic Diseases Branch for confirmatory diagnosis of parasitic infections. Following diagnosis, samples were de-identified and frozen in 200 μL aliquots at − 80 °C for use in assay development and validation. Samples containing *P. falciparum*, *P. vivax*, *P. malariae*, *Plasmodium ovale*, *B. microti*, and *Babesia divergens* were acquired in this way. For some rare blood-borne parasites, either animal blood or human blood samples collected during previous research studies and stored at − 80 °C were used. Bioinformatic analysis indicated that the restriction enzyme cut sites and the region of amplification were identical to human for all relevant animal samples. Some rare blood parasites that could not be acquired as true clinical samples were recreated by spiking uninfected human blood with cultured parasites—*L. donovani* subspecies *infantum*, *L. donovani* subspecies *donovani*, *T. cruzi*, and *T. brucei* cultures were added to whole human blood at a ratio of 1:10. All blood samples were collected into EDTA anticoagulant except for the clotted blood *W. bancrofti* sample. The full details of source, matrix, parasite identification, and DNA extraction methods are provided in Table 1.

### Assay design

In an effort to design universal parasite primers, Geneious bioinformatics software (Biomatters Inc., Newark, NJ, USA) was used to create an alignment of the 18S ribosomal RNA genes from the publicly available sequences of 24 protozoa, 17 helminths, and *Homo sapiens*. No *18S rRNA* sequences for *M. perstans* were available for inclusion in the alignment. Restriction enzyme cut sites were analyzed bioinformatically, and 18 primers were designed and tested in 14 primer combinations with 6 candidate restriction enzymes against a panel of four protozoan and one nematode, one trematode and two cestode helminth parasites (*P. falciparum*, *Toxoplasma gondii*, *T. brucei*, *C. felis*, *Brugia pahangi*, *Schistosoma mansoni*, *Dipylidium caninum*, and *Taenia* sp., relatively). From these analyses, one primer set was selected that amplifies an approximately 200 bp region of the 18S rRNA gene in all parasites tested, yielding a clearly visible band on a 1.5% agarose gel. Of the six restriction enzymes tested, BamHI-HF and XmaI (NEB, Ipswich, MA, USA) consistently yielded the highest degree of host DNA removal when paired with the selected primers.

### Universal parasite detection assay

Aliquots of parasite-positive human blood samples at a volume of 200 μL were spiked 1:100 with *C. felis*-infected cat blood at a concentration of 1.7 million parasites per microliter. DNA was extracted using the QIAamp DNA Blood Mini Kit (Qiagen, Redwood City, CA, USA) and eluted into 50 μL of PCR-grade water. Following extraction, DNA concentrations were determined using a Qubit 2.0 Fluorometer with the Qubit dsDNA High Sensitivity Assay Kit (Life Technologies, Grand Island, NY, USA). One hundred fifty nanograms of DNA were subsequently digested via incubation with 20 units of BamHI-HF and 20 units of XmaI in 1X CutSmart Buffer (NEB) in a final volume of 50 μL for 2 h in a 37 °C water bath. Digested samples were divided into two equal volumes, and enzymes and buffers were subsequently removed using the Monarch PCR & DNA Cleanup Kit (NEB). The Monarch kit enables template size selection (protocol dependent), and since there is uncertainty as to what the template size would be post-digestion, in most cases one sample aliquot was cleaned as a > 2 kb sample, and the second was cleaned as a < 2 kb sample according to the manufacturer’s instructions. Both samples were then eluted in 10 μL elution buffer (NEB).

PCR was performed with the cleaned and digested samples in a reaction volume of 20 μL using Q5 High-Fidelity DNA Polymerase (NEB) according to the manufacturer’s instructions and supplementing with Q5 High GC Enhancer. The primer sequences are as follows: CCGGAGAGGGAGCCTGAGA (forward) and GAGCTGGAATTACCGCGG (reverse). Samples were denatured at 98.0 °C for 2 min followed by 30 cycles of 98.0 °C for 10 s, primer annealing at 67.0 °C for 30 s and extension at 72.0 °C for 45 s, and a final extension at 72.0 °C for 5 min. Following PCR, samples were analyzed on a 1.5% agarose gel, cleaned with the Monarch PCR & DNA Cleanup Kit (NEB, < 2 kb), diluted 1:5 in elution buffer, and transferred to a 96-well plate for library preparation and sequencing.

Library preparation and sequencing were performed by the CDC Biotechnology Core Facility’s Genome Sequencing Lab using the NEBNext Ultra DNA Library Prep Kit for Illumina (NEB), and multiplexing was performed using the NEBnext Multiplex Oligos for Illumina Index kit (NEB) or using TruSeq HT Adapter sets (Illumina). No more than 80 samples were multiplexed on a single MiSeq run to ensure sufficient and consistent sequencing depth for each sample. Runs were prepared using a MiSeq Reagent Nano Kit v2 (PE250bp) (Illumina), and sequencing was performed on the Illumina MiSeq platform (Illumina). For all experiments, the paired digested and undigested (otherwise identical) samples were multiplexed on the same sequencing run to ensure they could be compared directly.

### Bioinformatic analysis

Geneious bioinformatics software (www.geneious.com) was used to analyze the raw fastq files generated by the MiSeq sequencing runs. Reads were paired and subsequently trimmed using the BBDuk plugin with a minimum quality of 35 and a minimum read length of 150 base pairs. Paired and trimmed reads were then mapped to the reference alignment described below using a minimum mapping quality of 35, a minimum overlap of 150, an allowance of zero mismatches per read, and a minimum overlap identity of 99%. To allow comparisons to be made between experiments and to assess the impact on restriction enzyme treatment for host template reduction, samples were normalized by dividing the species-specific mapped reads by the total paired reads in the sample, multiplying that value by 1000, and reporting the final value as reads per thousand. To account for index cross-talk between DNA samples multiplexed on the same Illumina run, a series of careful analyses were conducted to establish both a minimum cutoff to be applied uniformly across all samples as well as a shifting maximum cutoff to be applied on a sample-by-sample basis. Satisfaction of both criteria, as described below, would eliminate the occurrence of false-positive results.

### Reference alignment

Geneious bioinformatics software was used to establish a set of blood parasite reference sequences from among the 18S rRNA gene sequences found on GenBank. This reference database was used for assay development and validation and utilized the parasite GenBank accession numbers listed in Table 1 and the *Homo sapiens* sequence available under GenBank accession number HUMRGE. Since the region of amplification shares 100% sequence identity for *L. d. donovani*/*L. d. infantum*, *T. b. rhodesiense*/*T. b. gambiense*, and *W. bancrofti*/*L. loa*, only one representative sequence was included for each of these pairs in the final reference alignment cohort.

### Establishment of a minimum and maximum coverage cutoff value for positivity

A minimum coverage cutoff value of 20 reads was established for the specific protocol described in this study (i.e., using the Illumina MiSeq platform and a 500 cycle Nano Kit, and multiplexing 60 to 80 samples per sequencing run). The following formula was used to determine the minimum coverage cutoff:

$$ \left[\left({\mu}_{\mathrm{contam}\_\mathrm{all}}\right)+4(S.D.)\right]\times {\upmu}_{\mathrm{sample}\_\mathrm{reads}}={\mathrm{CUTOFF}}_{\mathrm{min}} $$

where *μ*_contam_all_ = the mean proportion of contaminating reads (i.e., the mean proportion of reads from all blood negative samples, *n* = 18, from this study that mapped to a parasite reference sequence), S.D. = standard deviation for *μ*_*contam*_ (four standard deviations above the mean was selected as the number of reads obtained for all samples represents a normal distribution), and *μ*_sample_reads_ = the mean number of reads obtained for each sample from 425 sequenced samples.

For this study, the minimum cutoff was calculated empirically and as follows (using all blood negatives in a sample):

$$ \left[(0.0001)+4(0.00026)\right]\times 17,553=19.855\ \mathrm{reads} $$

Consequently, specimens were only considered positive if more than 20 Illumina reads were detected for any given parasite species. This represents the maximum number of contaminating reads you might expect for any given specimen regardless of the parasite species under investigation, using this specific protocol.

Similarly, the maximum sliding cutoff was calculated for each individual sample using the following formula:

$$ \left[\left({\mu}_{\mathrm{run}\_\mathrm{contam}}\right)+4(S.D.)\right]\times {S}_{\mathrm{reads}}={\mathrm{CUTOFF}}_{\mathrm{max}} $$

where *μ*_run_contam_ = the mean proportion of contaminating reads within the negative control samples included in this specific sequencing run (at least four negatives were included in each run), S.D. = standard deviation for *μ*_run_contam_, and *S*_reads_ = the number of reads sequenced for the sample.

These coverage cutoffs take into account the index cross-talk (i.e., sample bleeding) for samples containing parasite reads that were multiplexed on the same sequencing run [25]. Two examples are provided below for calculating CUTOFFmax:

- $$ \left[(0.0002)+4(0.00033)\right]\times \mathrm{10,410}=15.823\ \mathrm{reads} $$
- $$ \left[(0.0001)+4(0.00023)\right]\times 24,204=24.688\ \mathrm{reads} $$

In example A, the sliding maximum rule would suggest a cutoff of 16 reads. However, at this cutoff we cannot be confident that this is not due to index cross-talk [25]. Consequently, we use the value of CUTOFF_min_ (20 reads) to exclude false positive results. In example B, CUTOFF_max_ is used (25 reads) to account for the fact that as the number of reads increases (i.e., depth) the proportion of contaminating sequences will also increase. Cutoff values are always rounded up to the nearest whole number.

This dual criterion system was developed to account for certain variables that may affect the sequencing output. As discussed above, at greater sequencing depth, it is more likely that contaminating reads will be detected; the sliding CUTOFF_max_ accounts for this. Additionally, the composition of samples sequenced in a given run will have an impact on the number and composition of contaminating reads present in negative control specimens as a result of index cross-talk. For example, if a run contains a large number of samples that are positive for *P. falciparum*, yet a small number of *Leishmania* positive samples, one would expect to see a larger proportion of *P. falciparum* reads in the negative control specimens compared to *Leishmania* reads. This is also the reason why the sliding CUTOFF_max_ is calculated for each run and for each parasite species individually—it compensates for the diversity of specimens that may be included within and between runs. Furthermore, if a single specimen out of 80 included on a single MiSeq run contains *P. knowlesi* DNA while all other samples are negative, it is possible that no *P. knowlesi* reads will be detected in the negative samples. In this case, the sliding CUTOFF_max_ cannot be used so CUTOFF_min_ is implemented.

### Multiple sequence alignments

All multiple sequence alignments were performed using the MUltiple Sequence Comparison by Log-Expectation (MUSCLE) algorithm. GenBank accession numbers for human parasites can be found in Table 1, those for vertebrates in Additional file 6: Table S1 and those for fungal organisms in Additional file 8: Table S2.

### Assessment of host DNA removal by restriction enzyme treatment

A dilution series was prepared from DNA extracted from the buffy coat layer of whole blood provided by healthy human volunteers and parasite DNA from 3D7 *P. falciparum* cultures. Samples were spiked with cat blood infected with *C. felis* prior to DNA extraction. Dilutions of human DNA were prepared at 3, 2.5, 2, 1.5, 1, 0.5, and 0 ng/μL and supplemented with 0.2 ng/μL DNA from a 3D7 *P. falciparum* culture (DNA equivalent of ~ 8600 parasite per microliter). Samples were then processed as described above by restriction digestion, PCR enrichment, and deep sequencing. Each digested sample was paired with an identical sample that was not restriction digested. The resulting sequencing reads were mapped against the reference database for quantification of parasite-derived reads. Note that for every experiment, an unquantified proportion of human reads was contributed by human products in the *P. falciparum* 3D7 culture.

### Limit of detection

For analysis of assay limit of detection, frozen samples of *P. knowlesi* from a non-human primate infection for which parasitemia had previously been determined by microscopy at the CDC (~ 144,000 parasites per microliter) were serially diluted in parasite-free whole blood. Samples were processed and sequenced as described above, in triplicate, with restriction digested samples paired with an identical undigested sample sequenced on the same run.

### Sample acquisitions

*P. knowlesi* in rhesus macaque blood was generously provided by Amy Kong (CDC, Malaria Branch, Atlanta, GA, USA); *Babesia duncani* in gerbil blood and *B. divergens* stabilate in human blood were kindly provided by Henry Bishop (CDC, Parasitic Diseases Branch, GA, USA); *B. malayi* microfilariae in feline blood were provided by Andy Moorhead (Filariasis Resource Reagent Resource Center (FR3), Athens, GA, USA); *W. bancrofti* microfilariae in human blood was provided by Patrick Lammie (CDC, Parasitic Diseases Branch, Atlanta, GA, USA); and *C. felis* in feline blood was provided by David Peterson (University of Georgia, Athens, GA, USA). *L. d. infantum*, *L. d. donovani*, and *T. cruzi* in culture were generously provided by Marcos deAlmeida (CDC, Parasitic Diseases Branch, Atlanta, GA, USA), *T. b. rhodesiense* in culture was provided by Stephen Hajduk (University of Georgia, Athens, GA, USA). Finally, *L. loa* and *M. perstans* purified DNA was generously provided by Thomas Nutman (National Institutes of Health, Bethesda, MD, USA). Funding for this work was provided by the CDC Advanced Molecular Detection initiative.

## Additional files

Additional file 1: **Figure S1.** *18S rRNA* Nucleotide alignment showing primers designed to detect a region of the gene wherein XmaI and BamHI restriction enzyme cut sites are present only in in the human host sequence and not in any parasite sequences. (TIF 13161 kb)

Additional file 2: **Figure S2.** Scatterplots demonstrating human reads per thousand (x-axis) vs parasite reads per thousand (y-axis) for undigested samples (black), digested samples cleaned using the > 2 kb DNA cleanup protocol (red), and digested samples cleaned using the < 2 kb DNA cleanup protocol (blue). Plots demonstrate a shift in reads for all parasite species tested: (a) *P. falciparum*, (b) *P. vivax*, (c) *P. ovale*, (d) *P. malariae*, (e) *P. knowlesi*, (f) *B. microti*, (g) *B. divergens*, (h) *B. duncani*, (i) *L. infantum* subspecies *infantum*, (j) *L. infantum* subspecies *donovani*, (k) *T. cruzi*, (l) *T. brucei* subspecies *rhodesiense*, (m) *B. malayi*, (n) *W. bancrofti*, (o) *L. loa*, and (p) *C. felis*. (TIF 5182 kb)

Additional file 3: **Figure S3.** Skewed results for *W. bancrofti* and *L. loa* due to sample composition. *W. bancrofti* samples had been collected into vials lacking anticoagulant. Uneven distribution of microfilariae in the clotted samples led to variations in parasite DNA concentrations in each aliquot and inconsistent resultant reads (**a**). Nevertheless, reductions in human reads per thousand were consistent with other analyses at 1.5- to 2.5-fold (**b**) despite wide variations in parasite relative reads per thousand (**c**). Meanwhile, *L. loa* samples were provided as worm DNA. Because of the lack of human DNA background, *L. loa* reads per thousand were consistently high (**d**), and relative reads indicated no fold-change between undigested and digested samples (**e**). Data shown represents results for 3 biological replicate runs. (TIF 6651 kb)

Additional file 4: **Figure S4.** Mock digestion of human blood spiked with cultured 3D7 *P. falciparum*-parasites confirmed that there was no difference between the number of parasite reads detected between mock digested and undigested samples (shown here in units of parasite reads per thousand). Furthermore, for matched samples subjected to a true restriction digest, the number of parasite reads detected was significantly larger compared to the undigested and mock digested samples (1way ANOVA with Dunnett’s multiple comparisons test, *p* < 0.005, *n* = 3, mean ± SD). (TIF 1302 kb)

Additional file 5: **Figure S5.** 18SrRNA Nucleotide alignment showing primer binding sites and both the XmaI and BamHI restriction enzyme cut sites are conserved in assessed vertebrates, including many livestock, companion animals, rodents and birds. Differences in sequence are shown in color. (TIF 15201 kb)

Additional file 6: **Table S1.** Common name, scientific name and Genbank accession number of vertebrates tested in silico and found to be suitable candidates for host reduction by this universal blood parasite detection method. (DOCX 15 kb)

Additional file 7: **Figure S6.** *18S rRNA* Nucleotide alignment showing conservation of primer binding sites but not restriction enzyme cut sites in assorted clinically relevant fungi. Although primer binding sites are conserved in all fungal DNA sequences and the XmaI and BamHI restriction enzyme cut sites are present in the human sequence, neither cut site is found in any fungal organism tested, indicating this method may also have increased sensitivity for detecting fungi in eukaryotic hosts. (TIF 18499 kb)

Additional file 8: **Table S2.** Genbank accession numbers of fungi tested in silico and found to be suitable candidates for detection and identification by this universal blood parasite detection method. (DOCX 14 kb)

## Abbreviations

NGS: Next-generation sequencing
TADS: Targeted amplicon deep sequencing
