# Supplementary figures and images for: Restriction enzyme digestion of host DNA enhances universal detection of parasitic pathogens in blood via targeted amplicon deep sequencing
Source: Microbiome. 2018 Sep 17;6:164. doi: 10.1186/s40168-018-0540-2 (PMC6142370; doi:10.1186/s40168-018-0540-2)

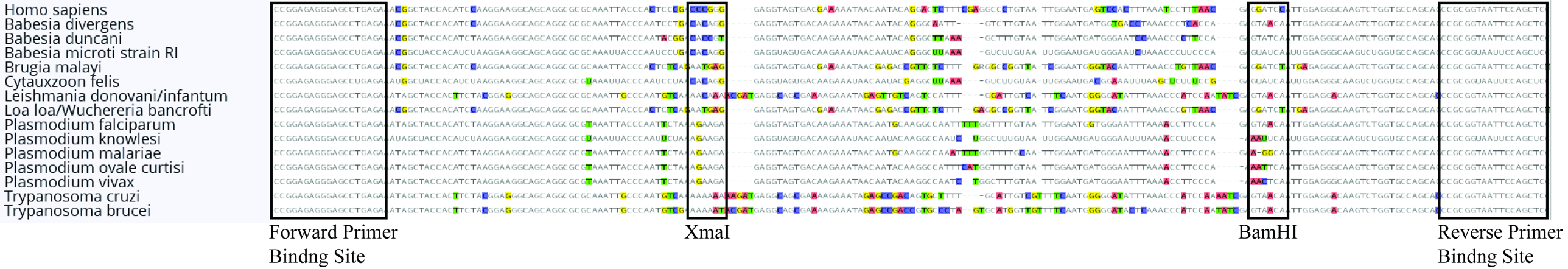

Supplement: Supplementary file 1 — Figure S1. 18S rRNA Nucleotide alignment showing primers designed to detect a region of the gene wherein XmaI and BamHI restriction enzyme cut sites are present only in in the human host sequence and not in any parasite sequences. (TIF 13161 kb) [file 40168_2018_540_MOESM1_ESM.tif]

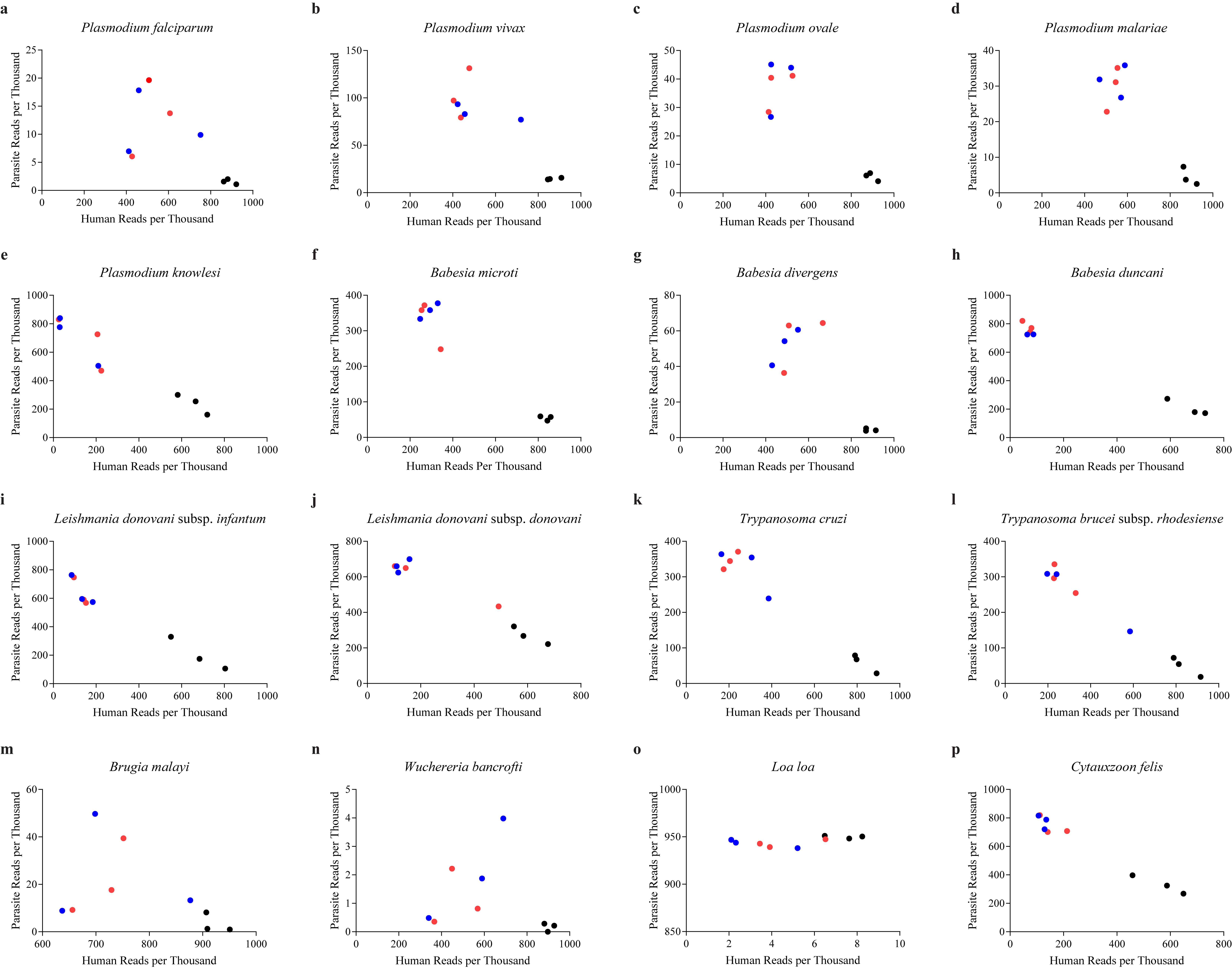

Supplement: Supplementary file 2 — Figure S2. Scatterplots demonstrating human reads per thousand (x-axis) vs parasite reads per thousand (y-axis) for undigested samples (black), digested samples cleaned using the > 2 kb DNA cleanup protocol (red), and digested samples cleaned using the < 2 kb DNA cleanup protocol (blue). Plots demonstrate a shift in reads for all parasite species tested: (a) P. falciparum, (b) P. vivax, (c) P. ovale, (d) P. malariae, (e) P. knowlesi, (f) B. microti, (g) B. divergens, (h) B. duncani, (i) L. infantum subspecies infantum, (j) L. infantum subspecies donovani, (k) T. cruzi, (l) T. brucei subspecies rhodesiense, (m) B. malayi, (n) W. bancrofti, (o) L. loa, and (p) C. felis. (TIF 5182 kb) [file 40168_2018_540_MOESM2_ESM.tif]

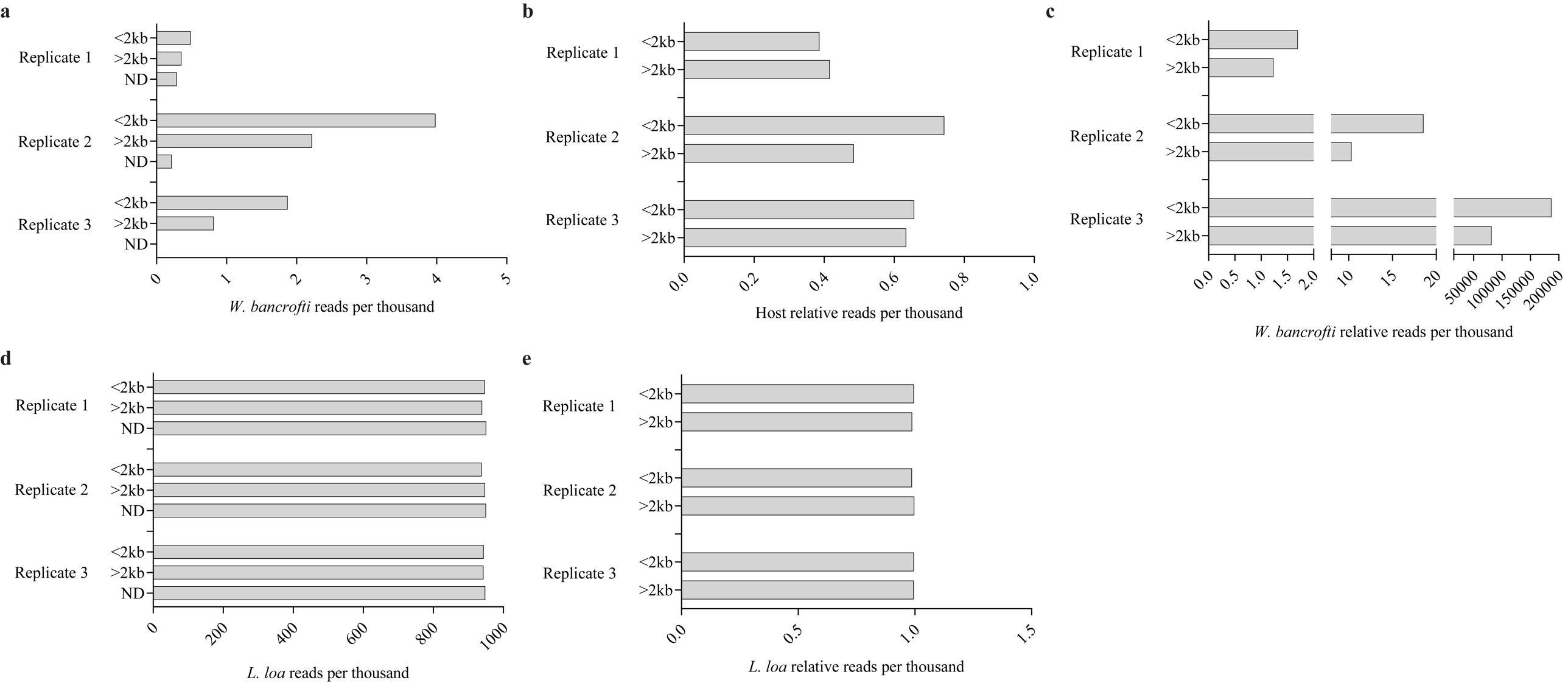

Supplement: Supplementary file 3 — Figure S3. Skewed results for W. bancrofti and L. loa due to sample composition. W. bancrofti samples had been collected into vials lacking anticoagulant. Uneven distribution of microfilariae in the clotted samples led to variations in parasite DNA concentrations in each aliquot and inconsistent resultant reads (a). Nevertheless, reductions in human reads per thousand were consistent with other analyses at 1.5- to 2.5-fold (b) despite wide variations in parasite relative reads per thousand (c). Meanwhile, L. loa samples were provided as worm DNA. Because of the lack of human DNA background, L. loa reads per thousand were consistently high (d), and relative reads indicated no fold-change between undigested and digested samples (e). Data shown represents results for 3 biological replicate runs. (TIF 6651 kb) [file 40168_2018_540_MOESM3_ESM.tif]

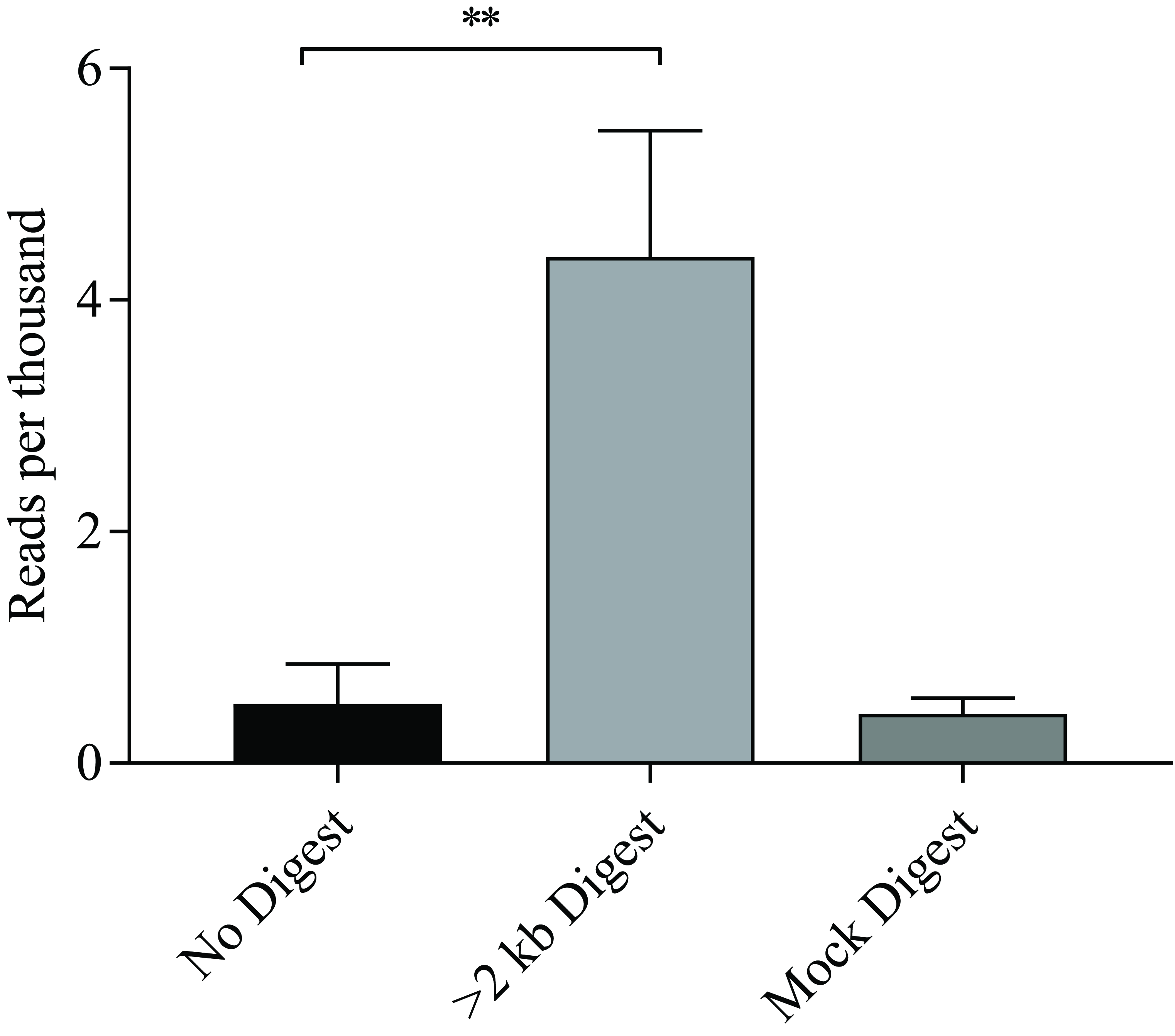

Supplement: Supplementary file 4 — Figure S4. Mock digestion of human blood spiked with cultured 3D7 P. falciparum-parasites confirmed that there was no difference between the number of parasite reads detected between mock digested and undigested samples (shown here in units of parasite reads per thousand). Furthermore, for matched samples subjected to a true restriction digest, the number of parasite reads detected was significantly larger compared to the undigested and mock digested samples (1way ANOVA with Dunnett’s multiple comparisons test, p < 0.005, n = 3, mean ± SD). (TIF 1302 kb) [file 40168_2018_540_MOESM4_ESM.tif]

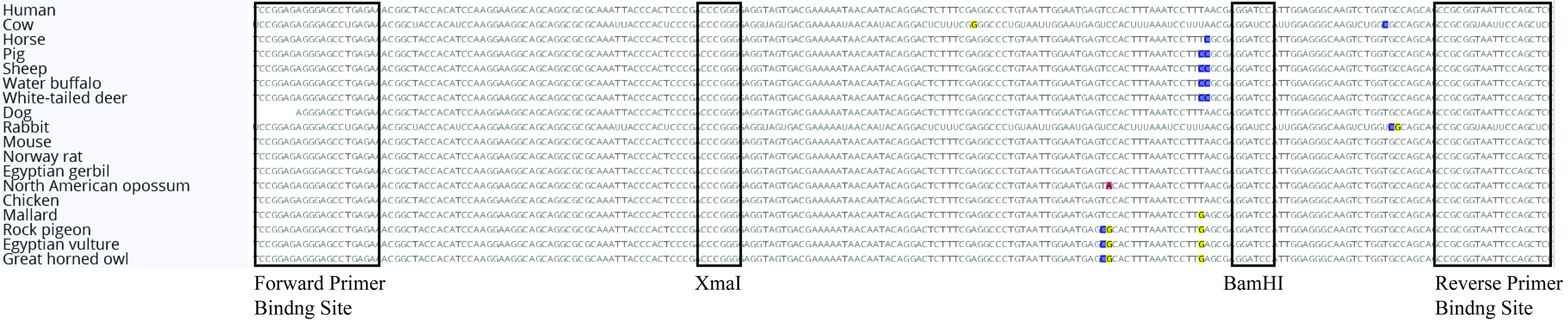

Supplement: Supplementary file 5 — Figure S5. 18SrRNA Nucleotide alignment showing primer binding sites and both the XmaI and BamHI restriction enzyme cut sites are conserved in assessed vertebrates, including many livestock, companion animals, rodents and birds. Differences in sequence are shown in color. (TIF 15201 kb) [file 40168_2018_540_MOESM5_ESM.tif]
