# Supplementary material for: Restriction enzyme digestion of host DNA enhances universal detection of parasitic pathogens in blood via targeted amplicon deep sequencing
Source: Microbiome. 2018 Sep 17;6:164. doi: 10.1186/s40168-018-0540-2 (PMC6142370; doi:10.1186/s40168-018-0540-2)
Supplement: Supplementary file 6 — Table S1. Common name, scientific name and Genbank accession number of vertebrates tested in silico and found to be suitable candidates for host reduction by this universal blood parasite detection method. (DOCX 15 kb) [file 40168_2018_540_MOESM6_ESM.docx]

**Table S1.** Common name, scientific name and Genbank accession number of vertebrates tested *in silico* and found to be suitable candidates for host reduction by this universal blood parasite detection method.

| **Common Name** | **Scientific Name** | **GenBank Accession #** |
| --- | --- | --- |
| Human | *Homo sapiens* | HUMRGE |
| Cow | *Bos taurus* | NR_036642 |
| Horse | *Equus caballus* | AJ311673 |
| Pig | *Sus scrofa* | AY265350 |
| Sheep | *Ovis aries* | KY129860 |
| Water buffalo | *Bubalus bubalis* | JN412502 |
| White-tailed deer | *Odocoileus virginianus* | EU823286 |
| Dog | *Canis lupus familiaris* | AY623831 |
| Rabbit | *Oryctolagus cuniculus* | NR_033238 |
| Mouse | *Mus musculus* | X00686 |
| Brown rat | *Rattus norvegicus* | RATRGEA |
| Egyptian gerbil | *Gerbillus gerbillus* | EU139317 |
| North American opossum | *Didelphis virginiana* | AJ311677 |
| Chicken | *Gallus gallus* | AF173612 |
| Mallard | *Anas platyrhynchos* | AF173614 |
| Rock pigeon | *Columba livia* | AF173630 |
| Egyptian vulture | *Neophron percnopterus* | AF173633 |
| Great horned owl | *Bubo virginianus* | AF173620 |
