# Supplementary material for: Restriction enzyme digestion of host DNA enhances universal detection of parasitic pathogens in blood via targeted amplicon deep sequencing
Source: Microbiome. 2018 Sep 17;6:164. doi: 10.1186/s40168-018-0540-2 (PMC6142370; doi:10.1186/s40168-018-0540-2)
Supplement: Supplementary file 8 — Table S2. Genbank accession numbers of fungi tested in silico and found to be suitable candidates for detection and identification by this universal blood parasite detection method. (DOCX 14 kb) [file 40168_2018_540_MOESM8_ESM.docx]

**Table S2** Genbank accession numbers of fungi tested *in silico* and found to be suitable candidates for detection and identification by this universal blood parasite detection method.

| **Scientific Name** | **GenBank**  **Accession #** |
| --- | --- |
| *Alternaria alternata* | KX494864 |
| *Aspergillus fumigatus* | FJ840490 |
| *Aspergillus niger* | HQ397705 |
| *Aspergillus terreus* | DQ173743 |
| *Candida albicans* | XR_002086442 |
| *Candida dubliniensis* | X99399 |
| *Candida glabrata* | AB094140 |
| *Candida tropicalis* | EU034726 |
| *Coccidioides immitis* | X58571 |
| *Cryptococcus neoformans* | HQ596559 |
| *Cryptococcus gattii* | EU402430 |
| *Fusarium solani* | AB473810 |
| *Fusarium oxysporum* | AB521041 |
| *Fusarium verticillioides* | XR_001989353 |
| *Histoplasma capsulatum* | X58572 |
| *Penicillium chrysogenum* | KU559908 |
| *Pneumocystis jirovecii* | XR_001936352 |
| *Saccharomyces cerevisiae* | Z75578 |
| *Scedosporium prolificans* | LPU43910 |
